# Method of Recognising the Presence of Blood on Clothes

**Published:** 1848-06

**Authors:** 


*Method of Recognising the Presence of Blood on Clothes.*—Fibrin has the property of attaching itself to the texture of clothes. Sulphuric acid has the property of dissolving textures made of hemp or linen without altering the fibrine. If, then, a texture of this sort is suspected of being stained with blood, it is to be plunged in concentrated sulphuric acid, which dissolves the texture, and leaves the fibrinous part of the blood presenting a network, where may be distinguished the impressions made by the texture on which the blood was fixed.—*Med. Gaz. from Journal de Chimie Médicale.*

